# Direct Structure–Performance Comparison of All‐Carbon Potassium and Sodium Ion Capacitors

**DOI:** 10.1002/advs.201802272

**Published:** 2019-04-24

**Authors:** Ziqiang Xu, Mengqiang Wu, Zhi Chen, Cheng Chen, Jian Yang, Tingting Feng, Eunsu Paek, David Mitlin

**Affiliations:** ^1^ Center for Advanced Electric Energy Technologies (CAEET) School of Materials and Energy University of Electronic Science and Technology of China Chengdu 611731 China; ^2^ Chemical & Biomolecular Engineering Clarkson University Potsdam NY 13699 USA

**Keywords:** lithium ion capacitors, potassium ion batteries, potassium ion capacitors, sodium ion batteries, sodium ion capacitors

## Abstract

A hybrid ion capacitor (HIC) based on potassium ions (K^+^) is a new high‐power intermediate energy device that may occupy a unique position on the Ragone chart space. Here, a direct performance comparison of a potassium ion capacitor (KIC) versus the better‐known sodium ion capacitor is provided. Tests are performed with an asymmetric architecture based on bulk ion insertion, partially ordered, dense carbon anode (hard carbon, HC) opposing N‐ and O‐rich ion adsorption, high surface area, cathode (activated carbon, AC). A classical symmetric “supercapacitor‐like” configuration AC–AC is analyzed in parallel. For asymmetric K‐based HC–AC devices, there are significant high‐rate limitations associated with ion insertion into the anode, making it much inferior to Na‐based HC–AC devices. A much larger charge–discharge hysteresis (overpotential), more than an order of magnitude higher impedance *R*_SEI_, and much worse cyclability are observed. However, K‐based AC–AC devices obtained on‐par energy, power, and cyclability with their Na counterpart. Therefore, while KICs are extremely scientifically interesting, more work is needed to tailor the structure of  “Na‐inherited” dense carbon anodes and electrolytes for satisfactory K ion insertion. Conversely, it should be possible to utilize many existing high surface area adsorption carbons for fast rate K application.

## 1 Introduction

There are primarily two types of commercial devices for electrochemical energy storage, batteries and supercapacitors (electrochemical capacitor, ultracapacitor). The former deliver high energy density, while the latter offer high power and high cyclability.^1^, ^2^ For instance, a commercial Panasonic NRC 18650 lithium ion battery (LIB) delivers a specific energy upward of 200 Wh kg^−1^, but with a maximum specific power being below 350 W kg^−1^.^3^ By contrast, a Maxwell K2 supercapacitor possesses specific power values as high as 10 kW kg^−1^, but with specific energies in the 5 Wh kg^−1^ range.^4^ An emerging target for advanced electrical energy storage devices is to deliver both high energy and high power in a single system.^1^, ^2^, ^5^ For this reason, battery–supercapacitor hybrid devices are attracting increasing scientific attention.^6^, ^7^, ^8^, ^9^, ^10^, ^11^, ^12^, ^13^, ^14^, ^15^, ^16^ A hybrid ion capacitor (HIC) is a relatively new device that is intermediate in energy between batteries and supercapacitors, while ideally offering supercapacitor‐like power and cyclability.^17^ One example of a potential end use of HICs is in regenerative braking applications, especially for subway trains, where relatively high energy and very fast charge capability are essential.^18^ The performance of an HIC is illustrated by a Ragone chart comparing its energy–power characteristics to a conventional electrical double‐layer capacitor (EDLC).^19^ As may be observed, such a device operates in the specific energy range of 200–60 Wh kg^−1^ by active material mass. This corresponds to a specific energy of roughly 50–15 Wh kg^−1^ by system mass, i.e., three to ten times higher than that of a state‐of‐the‐art supercapacitor. The specific power delivered is 200–20 000 W kg^−1^ by active material mass, which is high enough to obtain subminute charge–discharge times (e.g., 60 Wh kg^−1^ ÷ 20 000 W kg^−1^ = 11 s).

Although one may view HICs to represent the extreme end of high‐power ion batteries, the two voltage profiles are fundamentally different, with the former not containing plateaus.^13^ The voltage versus capacity profile of HICs is closer to that of a classical supercapacitor, i.e., nearly triangular without obvious plateaus.^20^ Importantly, HICs are fundamentally distinct from EDLC supercapacitors, as in the former charge storage includes bulk mechanisms. These bulk ion storage mechanisms are not fully understood, except that it would be impossible to achieve energies of 200–60 Wh kg^−1^ without them. The original embodiment of the hybrid ion capacitor operated in Li ions. The devices employed a standard intercalation battery graphite anode, combining it with an activated carbon (AC) cathode that stored charge by EDLC. However, since the electrodes are in series, the device power output was limited by Li intercalation into the micrometer‐scale graphite particulates. More effective recent versions design the structure of both electrodes to operate at high rates, markedly improving the overall device power characteristics.^11^, ^13^ Such architectures may be termed “intrinsically parallel,” providing enough rate capability in the anode to catch up to the performance of the cathode.

Sodium (Na) ion–based energy storage is attracting interest as a potentially lower‐cost alternative to Li ion systems, with readily available and geographically democratic reserves of the precursor. For this reason, materials for sodium ion battery (NIB, SIB) and sodium ion capacitor (NIC, SIC) anodes have recently received substantial attention.^21^, ^22^, ^23^, ^24^, ^25^, ^26^, ^27^, ^28^ Potassium (K)‐based energy storage is much newer than either Na or Li devices, and is beginning to attract attention as well.^29^ While Li is present in the Earth's crust at 20 ppm levels, Na and K are much more abundant at 23 000 and 17 000 ppm, respectively. Neither K nor Na reacts with aluminum, giving another price advantage over Li systems that require a copper current collector for the anode. In principle, K‐based devices will possess higher working voltages than Na‐based ones due to the redox potential of K^+^/K (−2.93 V vs standard hydrogen electrode (SHE)) that is lower than that of Na^+^/Na (−2.71 V vs SHE). For fast rate devices, K ions may also possess an advantage in terms of transport in electrolyte. The weaker Lewis acidity of K^+^ versus Li^+^ or Na^+^ should result in a lower Stokes radius of solvated ions, a higher transport number, and higher mobility.^30^

Recently, graphitic and amorphous carbon‐based potassium anodes have been explored.^31^, ^32^, ^33^, ^34^, ^35^, ^36^, ^37^, ^38^, ^39^, ^40^, ^41^, ^42^, ^43^, ^44^, ^45^, ^46^, ^47^, ^48^, ^49^, ^50^, ^51^, ^52^, ^53^, ^54^, ^55^, ^56^, ^57^, ^58^, ^59^, ^60^ Pure and heteroatom (e.g., N, O)‐doped carbons are regarded as highly promising candidates for large‐scale practical applications for K ion anode materials due to their low cost. However, due to the slower diffusion of K^+^ versus Li^+^ and even versus Na^+^, achieving fast charge and extended cyclability remains a major challenge. Although the role of heteroatoms in K carbon anode performance is not well understood, doped carbons generally display improved specific capacity, rate capability, and cyclability over conventional graphite. For instance, by nitrogen doping, the specific capacity of graphene may reach 350 mAh g^−1^ at 50 mA g^−1^, with 210 mAh g^−1^ remaining after 100 cycles.^61^ Liu et al. prepared nitrogen‐doped bamboo‐like carbon nanotubes, achieving 359 mAh g^−1^ at 100 mA g^−1^ and 186 mAh g^−1^ at 1000 mA g^−1^. Moreover after 1000 cycles at 500 mA g^−1^, the reversible capacity remained at 204 mAh g^−1^.^59^ Other recently reported heteroatom‐doped carbon materials include nitrogen‐doped carbon nanotubes, nanofibers, and nanosheets,^34^, ^49^ nitrogen‐doped graphene,^61^ nitrogen‐rich hard carbon (HC),^52^ nitrogen‐doped porous carbon,^62^ nitrogen/oxygen dual‐doped hard carbon,^37^ and sulfur/oxygen codoped porous hard carbon microspheres,^47^ all which show promising performance. Relevant studies include modeling of boron‐doped graphene^58^ and graphene‐like carbon nitride monolayer,^57^ as well as studies on polynanocrystalline graphite,^56^ hard–soft composite carbon,^53^ ultralight and flexible pencil trace,^51^ polycrystalline soft carbon semi‐hollow microrods,^50^ graphitic carbon nanocage,^43^ hyperporous sponge interconnected by hierarchical carbon nanotubes,^63^ engineering hollow carbon architecture,^46^ chemically bonded phosphorus/carbon composite,^45^ red phosphorus nanoparticle@3D interconnected carbon nanosheet framework composite,^44^ porous carbon nanofiber paper,^33^ dual‐graphite electrode,^40^ derived carbon from waste‐tire rubber,^35^ and short‐range ordered mesoporous carbon.^64^

There have also been a few very recent reports on nonaqueous potassium ion capacitors (KICs, PICs). Le Comte et al. reported symmetric AC devices in 1 mol L^−1^ tetraethylammonium tetrafluoroborate in acetonitrile, and graphite//AC asymmetric devices in 0.8 mol L^−1^ KPF_6_ in acetonitrile. The energy densities were in the 8–13 Wh kg^−1^ range with good cycling stability for the symmetric system.^65^ Fan et al. prepared a soft carbon//AC hybrid system with an energy density of 20–120 Wh kg^−1^ and a capacity retention of 71% after 1000 cycles.^66^ Dong et al. reported a K_2_Ti_6_O_13_//N‐doped nanoporous carbon hybrid system, which yielded 58.2 Wh kg^−1^ at 100 mA g^−1^ and survived 5000 cycles with a capacity retention of 75.5%.^67^ There has been a single comparison of K versus Na performance in carbons, which was specifically for battery anode applications, rather than for hybrid ion capacitor devices.^68^ There has also been a recent highly exciting study providing a direct sodium versus potassium comparison for full batteries, employing a co‐intercalation anode and an open framework cathode.^5^ These important but early results indicate that KICs are novel and must be understood further and in much greater detail.

Overall, much less is known about KICs as compared to the more studied NIC systems.^2^ The objective of this study is to provide the first direct performance comparison of symmetric and asymmetric carbon‐based HIC architectures operating using K versus Na ions. Two types of carbons are explored, being representative of K and Na electrode systems in the literature. Both are natural precursor‐derived materials. The first is a high‐performance dense hard carbon derived from leftover silkworm feed, namely, mulberry tree stems. It possesses an amorphous structure and bulk ion insertion mechanisms representative for what is employed for battery‐type ion anodes. The second is a N‐rich high surface area carbon that is derived from silk worm waste. It possesses the morphology and heteroatom content that is representative of ion adsorption–type cathodes employed for high‐rate applications. However, neither carbon has been explored for potassium ion battery or hybrid capacitor applications. The carbons, as well as the optimized devices, show state‐of‐the‐art performance. However, the main focus of this paper is not performance maximization per se. We also point out that this is not a “precursor” study, such as often the case in energy storage publications. The carbons employed are meant as model systems, the precursors being chosen to achieve the targeted structures. The aim of this study is to provide fundamental new insight regarding the suitability of “Na‐inherited” carbon materials and device architectures for emerging high‐power potassium applications. This objective has not been achieved in prior art but is critical for advancing the KIC field.

## 2 Results and Discussion

### 2.1 Microstructural Characterization

**Figure 1:**
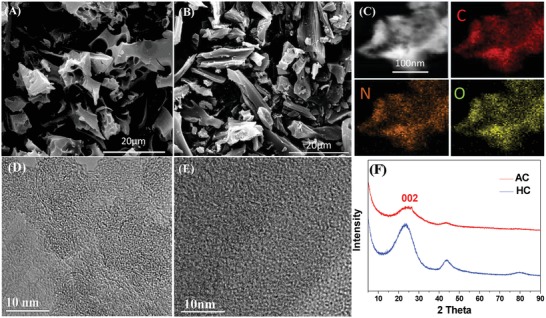
A) SEM image of activated carbon “AC”. B) SEM image of hard carbon “HC”. C) TEM HAADF image and EDXS analytical maps of C, N, and O for AC. D,E) HRTEM images highlighting the disordered structures of AC and HC, respectively. F) X‐ray diffraction patterns of AC and HC, highlighting their amorphous structure.

**Figure** **1**A,B shows scanning electron microscopy (SEM) images of AC and HC. Both materials are micrometer‐scale 3D particulates, on par in their scale with conventional battery graphites, hard carbons, and activated carbons. Figure 1C displays high‐angle annular dark‐field (HAADF) transmission electron microscopy (TEM) images and energy‐dispersive X‐ray spectroscopy (EDXS) elemental maps of C, N, and O elements in AC. The biological precursor is naturally rich in oxygen and nitrogen, which are retained following pyrolysis. Figure 1D presents a high‐resolution TEM (HRTEM) image of AC demonstrating the low degree of ordering in the material. As per HRTEM, the HC specimen is similarly disordered, being shown in Figure 1E. Figure 1F shows the X‐ray diffraction (XRD) patterns of AC and HC. The patterns show two broad diffraction peaks that are indexed as (002) and (100) of the pseudographitic domains.^69^ The average graphene interlayer spacing can be calculated from the center position of (002) peaks.

As **Table** **1** shows, the mean graphene layer spacing (*d*_002_) for both materials is significantly larger than that of graphite (0.40 and 0.39 nm vs 0.3354 nm). While in principle such dilated layer spacing may allow for ion intercalation into HC at the negative anode voltage, this is the case only for Na, but not for K. The average dimensions of the ordered graphene domains (*L*_a_, *L*_c_) are calculated by the well‐known Scherrer equation, using the full‐width at half‐maximum values of (002) and (100) peaks, respectively. As per Table 1, the average domain thickness *L*_c_ is on par for both carbons, being 1.50 nm for AC and 1.68 nm for HC. The average domain width *L*_a_ for HC is twice that for AC, being 8.04 nm versus 4.04 nm.

**Table 1:** HC and AC structural and textural properties

Sample	Carbon structure				Textural properties			
	*d*_002_ [nm]	*L*_a_ [nm]	*L*_c_ [nm]	*I*_G_/*I*_D_ ^a)^	*S*_BET_ ^b)^ [m^2^ g^−1^]	*V*_t_ ^c)^ [cm^3^ g^−1^]	Micropores [vol%]	Mesopores [vol%]
AC silkworm excrement (SE)	0.40	4.04	1.50	0.71	1602	1.04	81.9	18.1
HC mulberry stalk (MS)	0.39	8.04	1.68	0.97	32	0.05	52.2	47.8

^a)^ *I*_D_ and *I*_G_ are the integrated intensities of D and G bands, respectively

^b)^ Surface area was calculated with the BET method

^c)^ The total pore volume was determined at a relative pressure of 0.98.

**Figure 2:**
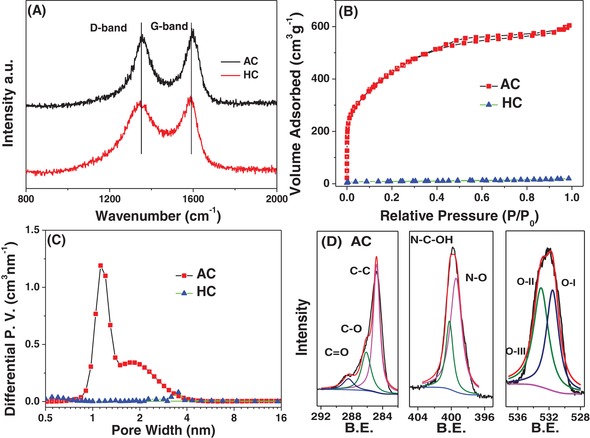
A) Raman spectra of AC and HC. B) Nitrogen adsorption isotherms of AC and HC. C) Pore size distribution of AC and HC. D) XPS C, N, and O spectra of AC. The survey spectra are shown in the Supporting Information.

Raman spectroscopy data for the two carbons are shown in **Figure** **2**A. Both AC and HC exhibit broad disorder‐induced D bands (≈1340 cm^−1^) and in‐plane vibration G bands (≈1580 cm^−1^). The values of the integral intensity of D and G bands may be employed to index the degree of defectiveness in a carbon. Figure S1 (Supporting Information) shows the actual fits of the Raman spectra of AC and HC. Table 1 shows the integrated intensity ratio of the G and D peaks for both materials. The *I*_G_/*I*_D_ ratio for HC is 0.97, while it is 0.71 for AC. In hard carbons, an integrated G to D band ratio equal to or greater than 1 is known to promote reversible Na intercalation at anode voltages.^19^ As will be demonstrated, the same HC material with K shows minimal electrochemical evidence of reversible intercalation even at relatively slow charging rates.

Figure 2B shows the nitrogen adsorption–desorption isotherms of AC and HC, while Figure 2C shows their pore size distributions (obtained by density functional theory (DFT)). Table 1 also provides the porosity characteristics of both materials. The AC specimens show type I/IV isotherms with a Brunauer–Emmett–Teller (BET) surface area of 1602 m^2^ g^−1^, 82% micropores, and 18% mesopores. The total pore volume was 1 cm^3^ g^−1^. As expected, the pyrolyzed but not activated HC possessed a relatively low surface area of 32 m^2^ g^−1^. Figure 2D and Figure S2 (Supporting Information) display the X‐ray photoelectron spectroscopy (XPS) fitted high‐resolution spectra and the survey spectra, respectively. The C 1s, N 1s, and O 1s of the SE are shown in Figure 2D, whereas the survey spectra and the C 1s and O 1s spectra are shown in Figure S2 (Supporting Information).

**Table** **2** lists the surface and bulk element composition of the prepared carbons, as well as the oxygen functionalities obtained by XPS. The nitrogen content is 11.66 wt% for AC, which is on the high end of reported K anode materials.^41^, ^59^, ^70^, ^71^, ^72^ The N 1s peak mainly includes pyridinic N and N oxides (399.5–402 eV).^19^ The C 1s peak is dominated by a C—C bond at 284.6 eV and the O 1s peak is at 531–533 eV.^73^ The oxygen content of AC was 17.87%, while that of HC was 8.24%. The difference in the heteroatom content may be rationalized by the N and O contents in the precursor. The N moieties should be highly chemically active and are likely to introduce additional defects into the graphene planes. Nitrogen functionalities and associated defects will enhance AC's capacity to reversibly bind with charge carriers such as Li, Na, and K. Excrement of most living things is naturally rich in nitrogen and oxygen, whereas wood is not. Other potential impurities that may be present in plant‐based precursors (e.g., P, K, Mg, and Ca) were below the detection limits of XPS analysis, being both volatilized during synthesis and further removed by the postsynthesis HCl wash.

**Table 2:** Bulk and surface chemistry of AC and HC

Sample	XPS [wt%]			EDXS [wt%]			Functionality [% of O 1s]		
	C	N	O	C	N	O	O‐I	O‐II	O‐III
AC	71.43	11.66	16.91	70.22	11.91	17.87	57.62	42.27	0.11
HC	89.08	1.68	9.24	90.17	1.59	8.24	40.26	58.35	1.39

### 2.2 Anode Performance Comparison

**Figure 3:**
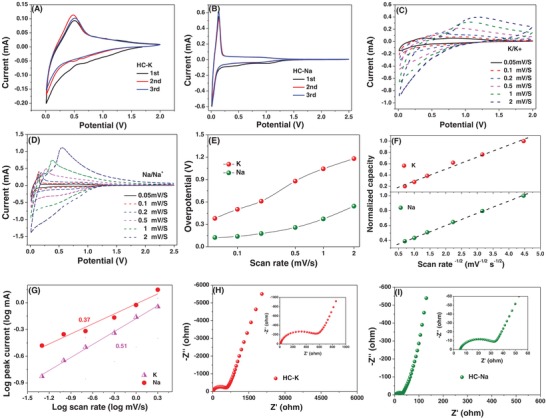
Half‐cell electrochemical performance of HC versus K/K^+^ and Na/Na^+^. A,B) CV tested at 0.1 mV s^−1^ for K/K^+^ and Na/Na^+^, respectively. C,D) Multirate CV curves versus K/K^+^ and Na/Na^+^, respectively. E) The overpotential values of the potassium and sodium systems at various scan rates. F) The variation of normalized capacity as a function of the inverse square root of the scan rate. G) *b*‐value determination based on logarithmic peak currents versus scan rate. H,I) EIS for K/Ka^+^ and Na/Na, respectively.

**Figure** **3**A–I contrasts the electrochemical performance results for HC tested in a half‐cell configuration versus K/K^+^ and Na/Na^+^. Figure 3A,B compares the cyclic voltammetry (CV) curves for K/K^+^ and Na/Na^+^ for cycles 1–3, tested at 0.1 mV s^−1^. During potassiation, there is a broad cathodic peak starting at near 1 V and continuing to the terminal 0.01 V. The anodic (depotassiation) peak being is centered at 0.5 V and shows a large hysteresis as compared to the cathodic peak. This hysteresis is markedly larger than that for the sodiation–desodiation reactions, with CV being shown in Figure 3B. Being partially ordered and with a dilated interlayer spacing, HC is able to reversibly intercalate Na ions with majority of the reversible capacity being below 0.25 V. The desodiation possesses a low hysteresis, the difference between the peak charge and discharge current in the CV being below 0.15 V at the slower scan rates, as per Figure 3E. Importantly, the low‐voltage plateau demonstrated in the CVs and the galvanostatic data for HC with Na is distinctly missing with the same materials tested against K.

With Na, there is now a large body of literature discussing the dominant charge storage mechanism below ≈0.25 V versus Na/Na^+^. For instance, the authors demonstrated through detailed X‐ray diffraction and related analysis that the reversible Na intercalation into partially ordered hard carbons is the key source of capacity below 0.25 V versus Na/Na^+^.^74^, ^75^ It was shown that with increasing graphene layer ordering and domain size, due to a higher heat treatment temperature, the low‐voltage plateau capacity increased while the higher‐voltage capacity either decreased or remained constant. Numerous follow‐up studies confirmed the low‐voltage Na intercalation mechanism into nongraphitic but partially ordered hard carbons.^69^ For instance, the authors employed Raman Spectroscopy analysis to provide further evidence of Na staging reactions in these partially ordered albeit nongraphitic domains of graphene.^2^ Judging from the broad CV curves and the highly sloping voltage profiles, it is doubtful that K undergoes the same intercalation process into the hard carbon as does Na. However, more detailed analysis is required to confirm or negate this conclusion. Important preliminary work has been done on this subject, although to date more analytical studies are necessary.^54^, ^55^, ^56^, ^61^ Practically speaking, having a highly sloping voltage profile in an anode places full K‐based HIC devices at an energy disadvantage since the average device voltage window is lower.

The presence of O in the HC material should be important for reversible adsorption of both Na and K ions at less negative anode voltages. Sodium reversibly binds to the heteroatom moieties, to the actual dopants, or to the defects in the structure that accompany dopant introduction.^69^ It is expected that O and N would have a similar influence on K storage. The most ion‐active oxygen moieties should be the quinone‐type groups (C=O/O—C=O, O—I type) due to the unsaturated carbon–oxygen double bond. The HC materials possess significant O—I content, as per Table 2. Researchers have reported that there is substantial charge storage capacity associated with reversible Na binding to this functional group.^16^, ^76^ Quinone and other functional groups' effect on K binding is not fully understood, although one can indirectly gather that the various oxygen groups should be a significant contributor to reversible capacity there as well. As per the CV curves and the galvanostatic data, reversible binding of K occurs at higher voltages than for Na. There is also a larger hysteresis. Both affects can be directly interpreted as being due to a higher binding energy between these sites and the K ions versus the Na ions. We argue that lower overall capacity of K versus Na is due to diffusional limitations of the former, which inhibits full potassiation even at relatively slow charging rates. As per Figure 3C, it may be observed that the potassiation overpotential at every scan rate tested is consistently higher than the sodiation overpotential. This difference increases with scan rate, being nearly 0.7 V at 2 mV s^−1^. We believe that this is also a diffusivity effect, where the inherently sluggish kinetics of K insertion–extraction in HC leads to major *IR* loss on both charge and discharge.

To further understand the electrochemical kinetics of HC with K versus Na, we plotted the current response at scan rates of 0.05–2 mV s^−1^. Such multirate CV scan tests are suited for examining charge/discharge kinetics to understand regions of diffusional versus reaction control.^77^ These results are shown in Figure 3C,D, comparing the K and Na systems in both cases. A well‐established method at looking at the onset of diffusional limitations is a mathematical “*b*‐value” analysis. This straightforward method tracks the transition in the time dependence of the peak current, i.e., the peak reaction rate. The current change with the scan rate may be expressed as *i* = *av^b^*, where *a* and *b* are adjustable constants. Having a *b*‐value of 0.5 signals a diffusion‐limited process. Conversely, a *b*‐value of 1 is for any activation polarization reaction. This may be for any reaction‐limited process, including but not limited to surface capacitance and pseudocapacitance. For example, nucleation‐controlled growth of a precipitate phase is a solid‐state process that follows linear time kinetics, but is not capacitive in nature. For micrometer‐scale low surface area materials such as HC, diffusional limitations are in the solid state rather than through pore‐filled electrolyte.

The relation between the normalization capacity during the CV tests and the *v* ^−1/2^ values is one way to understand at what rates reaction control transitions to diffusion control. These results are shown in Figure 3F. The normalized capacities are calculated according to *C* = *i*Δ*t*/*m*Δ*E*, where *C* is the capacity, Δ*t* and Δ*E* are the time and voltage changes around the peak, respectively, and *m* is the mass of electrodes. As per Figure 3F, at all scan rates tested both potassiation and sodiation reactions are diffusion controlled. A plot of the normalized capacity versus the inverse square root of scan rate remains linear. There is no low scan rate where the process transitions to reaction control instead. Figure 3G confirms this conclusion, where the *b*‐value analysis yields a square root time dependence at all scans. The results in Figure 3G are shown for the anodic scans only, since there was too much uncertainty in finding the maxima of the cathodic peaks. We do recognize that the kinetics of the cell are also affected by other factors that may be different in the two systems, such as the charge transfer kinetics at the anode and the ionic conductivity in the electrolyte.^78^, ^79^, ^80^

Figure 3H,I compares the electrochemical impedance analysis (EIS) of the half‐cells with K/K^+^ and Na/Na^+^, respectively. Figure 3H,I presents the high‐frequency portion of Nyquist plots of HC with K and Na, respectively. The associated equivalent circuit fits are shown in Figure S3 (Supporting Information). The corresponding full spectra are shown in Figure 3H,I, with the relevant sections identified by a rectangle. Analysis is shown after three cycles, tested at open‐circuit potential, in the fully depotassiated/desodiated state. The Nyquist plots contain a semicircle located at the high‐frequency region, which correlates to overlapped solid electrolyte interphase (SEI) impedance *R*_SEI_ and charge transfer impedance *R*_CT_. Although in principle the higher‐frequency *R*_SEI_ should be distinguishable from the lower‐frequency *R*_CT_, in practice they overlap for both Na and K. We label the semicircle *R*_SEI_ with the understanding that it also includes the charge transfer contribution. The intercept at high frequency with real axis is associated with electrolyte resistance (*R*_e_), although strictly speaking it also includes a summation of the Ohmic resistances of various portions of the cell. It may be observed from the plots that there is more than an order of magnitude difference between *R*_SEI_ for K (585 Ω) versus *R*_SEI_ for Na (23.4 Ω). The result indicates that the SEI structure of HC tested with K is much more resistive than that with Na. This would be another key contributor to the observed overpotential difference, and would lead to worse overall kinetics, as is demonstrated in the next set of figures.

**Figure 4:**
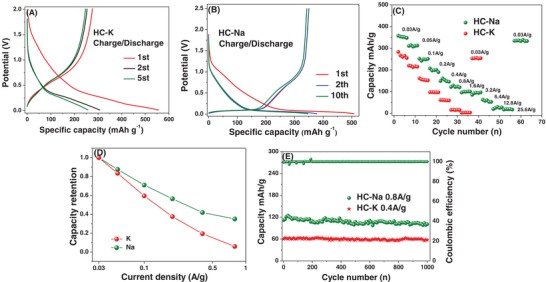
Half‐cell electrochemical performance of HC versus K/K^+^ and Na/Na^+^. A,B) Galvanostatic charge–discharge curves at 0.03 A g^−1^ for K/K^+^ and Na/Na^+^, respectively. C,D) Rate performance capacity retention comparison, showing the absolute capacity and the capacity retention fraction as a function of current density, respectively. E) Cycling capacity retention comparison.

**Figure** **4** compares the galvanostatic performance of HC in K versus Na half‐cells. These results are consistent with the CV data presented in the earlier set of figures. Figure 4A,B shows the actual galvanostatic charge–discharge curves at 30 mA g^−1^, for cycles 1, 2, and 5. Figure 4C,D compares the capacities at various current densities, with Figure 4C showing the absolute capacity and Figure 4D showing the relative capacity retention. Figure 4E compares the cycling stability of HC with K and Na. With K, the half‐cell displays a high‐voltage sloping region with a minimal low‐voltage plateau. This is distinct from the Na case, where there is nearly 200 mAh g^−1^ of reversible capacity below 0.1 V versus Na/Na^+^. This is equivalent to more than 57% of the total capacity being in the plateau region, i.e., attributed to reversible Na intercalation between graphene planes. For K, the low‐voltage sloping region of the capacity curve holds about 50 mAh g^−1^ of charge, which is only 17% of its total capacity. The overall reversible capacity with K is lower as well, being 270 mAh g^−1^ versus 349 mAh g^−1^. The cycle 1 Coulombic efficiency (CE) for K is also lower, being at 49% versus 69% for Na. At cycle 1, there is more irreversible K ion trapping, especially at the higher voltages. Reversible K ion storage also occurs at higher voltages than with Na. Based on the shape of the galvanostatic data, one can reasonably argue that reversible K ion binding at defect sites dominates capacity.

As per Figure 4C,D with increasing current density, the reversible capacity of the K cell decreases faster than that of the Na cell. For instance, at 1600 mA g^−1^, the K cell has negligible capacity, whereas the Na cell retains 100 mAh g^−1^. The poorer rate capability with K is due to solid‐state diffusional limitations and the influence of a higher combined SEI and interfacial impedance. Diffusion limitations, be they through the bulk carbon or through the SEI, will become most severe at high charging rates. Both the K and the Na cells exhibit a relatively stable cycling performance, as shown in Figure 4E. After 1000 cycles, the capacity retention of with K is 85% at 400 mA g^−1^. The capacity retention with Na is 80% at 800 mA g^−1^. We consider these cycling retention values to be on par. However, the origin of the capacity decay may be different with Na versus K. For Na intercalating into HC, the associated volume changes lead to capacity decay due to gradual cycling‐induced exfoliation. Since K does not significantly insert into HC, the capacity decay may be instead driven by excessive SEI formation, agreeing with the EIS results.

### 2.3 Device and Cathode Performance Comparison

When demonstrating device performance, the half‐cell results are normally presented ahead of the full‐cell device data. While this is a practical approach that at this point is established, it is important to realize some fundamental differences between half‐cell and full‐cell results. Half‐cell results highlight the performance versus K, Na, or Li metal counter electrode, which is also the reference. In a half‐cell, the voltage swing of the working electrode is set. Hence, the thermodynamic conditions of the working electrode and of the electrolyte are well defined at every current. However, in a full‐cell hybrid device, the electrolyte may not see any metal interface at all, unless there is unintended plating on the anode during cycling. In a full cell, the relative voltage swing of each electrode, as a fraction of the total voltage, is determined by the anode‐to‐cathode capacity ratio. In a half‐cell, the HC electrode will develop an SEI below ≈1 V versus K/K^+^, Na/Na^+^, or Li/Li^+^. Irreversible capacity loss associated with SEI formation may be quite substantial with initial CE values being as low as 30%.^78^ The metal counter electrode itself is highly catalytic toward SEI formation, showing up the EIS spectra, etc. Conversely, in AC–AC and HC–AC full cells, there should be no metal K/Na in the device (unless plating occurs). The active ions originate from the dissociated salt. Therefore, the two sets of data for a given material are not directly transposable. For example, electrolyte decomposition on the anode in a full cell cannot be directly predicted from half‐cell performance. Instead, SEI formation must be obtained directly from full‐cell results.

We investigated two types of architectures for both K and Na devices. The first is an approach based on symmetric AC–AC cells. (Strictly speaking, the configuration is not truly “symmetric” since the mass ratio between the anode and the cathode is 1:2, so as to achieve capacity balancing.) This approach borrows from the ultracapacitor literature where it is the norm to employ an identical material for both electrodes. The core difference for hybrid device is that when employing battery electrolytes, there are other charge storage mechanisms in addition to EDLC. A symmetrical‐like NIC configuration is known to give promising energy and cyclability values as long as the device voltage window is kept narrow enough to prevent excessive SEI formation on the anode.^8^ A conventional acetonitrile solvent used for EDLC devices usually has a 2.7 V maximum voltage window. Carbonate electrolytes used for NICs and KICs should be thermodynamically stable at 3 V, especially if there are no catalytic metal surfaces. Therefore, there is an inherent advantage since device energy scales with the voltage window squared. What we observed was that with both K and Na, the stable voltage window for a symmetric AC–AC device was 3 V. At higher voltages, capacity decay was rapid. This is likely due to both SEI formation on the anode and cathode–electrolyte interface formation on the cathode. However, with both K and Na, the 3 V AC–AC devices were able to cycle relatively well. It is important to emphasize again that a device voltage of 0 V (fully discharged) is not synonymous with a half‐cell voltage of 0 V versus Na/Na^+^ or K/K^+^. In the half‐cell, the electrolyte is thermodynamically unstable while being in direct contact with a catalytic metal surface.

In an AC–AC hybrid device configuration, despite the electrodes being the same material, the charge storage mechanisms will be fundamentally distinct. As discussed, both K^+^ and Na^+^ are able to insert into the bulk of most anode carbons. This is fundamentally different from an ideal EDLC ultracapacitor, where there is no bulk cation insertion. It is also the origin of the nonideality of the hybrid device charge–discharge curves, since pure physical adsorption would yield perfectly triangular profiles. Conversely, the ClO_4_^−^ and PF_6_^−^ counterions should only physically adsorb onto the AC cathode surfaces. Bulk ClO_4_^−^ and PF_6_^−^ insertion has not been reported. Therefore, for KICs and NICs, reversible adsorption of PF_6_^−^ and ClO_4_^−^ counterions on the cathode will also contribute to the reversible capacity. Since Na^+^ and K^+^ are naturally adsorbed on carbon surfaces at open circuit, another source of capacity in the cathodes should be their repulsion during positive polarization. Unfortunately, the body of experimental and theoretical knowledge related to the cathode charge storage mechanisms in both NICs and KICs is much narrower than that for the anodes. More work is needed to understand the high‐voltage cathode‐side charge storage mechanisms in high surface area carbons for both types of devices.

**Figure 5:**
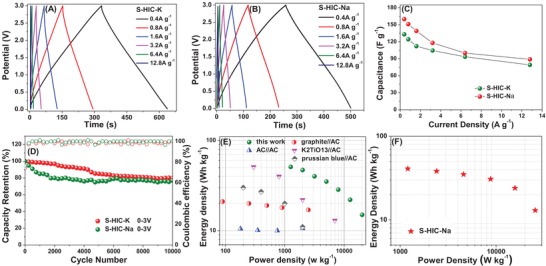
Performance comparison of S‐HIC device based on K^+^ and Na^+^. A,B) Charge–discharge curves at different current densities. C) Specific capacitance of SE versus current density. D) Extended cycling performance, tested at 0.8 A g^−1^. E) Ragone plot comparison of S‐HIC‐K versus prior HIC‐K literature. F) Ragone plot comparison of S‐HIC‐Na.

The electrochemical performance results of AC–AC potassium‐ and sodium‐based HICs are shown in **Figure** **5**. Figure 5A,B highlights the galvanostatic results for symmetric HIC based on K (S‐HIC‐K) and symmetric HIC based on Na (S‐HIC‐Na). It can be seen that the shape of charge–discharge curves deviates from the ideal triangular due to the non‐EDLC storage mechanisms described earlier. Tests were performed at the same range of current densities for S‐HIC‐K and S‐HIC‐Na, 0.4–12.8 A g^−1^. As per Figure 5C, the performance of S‐HIC‐K is on par with S‐HIC‐Na in terms of energy and rate capability. The cycle 1 CE of the S‐HIC‐K and S‐HIC‐Na devices is also similar, being 74% and 76%, respectively. The Nyquist EIS plot shown in Figure S4 (Supporting Information) indicates that the S‐HIC‐K shows a somewhat lower *R*_CT_ versus S‐HIC‐Na, being 2.9 Ω versus 5.2 Ω. As per Figure 5D, the shorter‐term cyclability of S‐HIC‐K is somewhat superior to S‐HIC‐Na, although after 10 000 cycles the two systems also become on par.

Figure 5E compares the Ragone chart characteristics of the S‐HIC‐K devices versus recent asymmetric K ion capacitor and K‐based supercapacitor (AC//AC) literature. This comparison is only meant to illustrate the general literature trends and is not meant to promote the current system as “superior,” etc. To date, there are only a handful of full K ion device publications and a more comprehensive data set is definitely needed before meaningful energy–power comparisons are possible. One key point, however, that is illustrated by Figure 5E is that K‐based cells that employ bulk insertion anodes all appear to be power limited. Comparing the Ragone chart characteristics of S‐HIC‐K in Figure 5E with the identically tested S‐HIC‐Na in Figure 5F, it is evident that the K device delivers analogous specific energies at all specific powers tested. For instance, AC–AC‐K achieved 51 Wh kg^−1^ at 1260 W kg^−1^, 35 Wh kg^−1^ at 5020 W kg^−1^, and 15 Wh kg^−1^ at 20 110 W kg^−1^. The AC–AC‐Na achieved 41 Wh kg^−1^ at 1200 W kg^−1^, 35 Wh kg^−1^ at 4576 W kg^−1^, and 13 Wh kg^−1^ at 25 600 W kg^−1^. We consider these values similar, indicating the kinetics of Na and K diffusion in electrolyte‐filled pores, as well as the reversible adsorption on carbon surfaces, are on par as well.

The K and Na devices were also tested in an asymmetric hybrid ion capacitor configuration, namely, employing the low surface area bulk HC anode opposing the high surface area AC. These devices are labeled A‐HIC‐K and A‐HIC‐Na, i.e., asymmetric hybrid ion capacitors. We employed a voltage window commonly employed for hybrid Na and Li devices (1.5–4.2 V). This range maximized the operating voltage window without decomposing the electrolyte. However, without the presence of catalytic Na/K bulk metal, a voltage window of 0–2.7 V would have achieved the same effect. Upon positive polarization, the AC electrode will reversibly adsorb ClO_4_^−^ and reversibly release Na^+^. The capacity in AC is achieved both by EDLC of ClO_4_^−^ and through an interaction of Na^+^ with surface defects and oxygen functionalities. One could argue a similar process with AC employed for A‐HIC‐K: Upon polarization, K^+^ is reversibly released and PF_6_^−^ is reversibly adsorbed. Capacity is achieved both by EDLC of PF_6_^−^ and through interaction of K^+^ with surface defects and oxygen functionalities.

**Figure 6:**
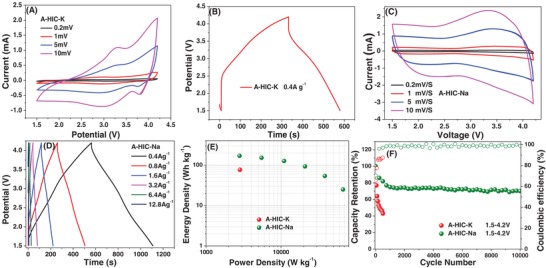
Performance comparison of an A‐HIC device based on K^+^ and Na^+^. A) CV curves of A‐HIC‐K recorded at different scan rates. B) Galvanostatic curves of A‐HIC‐K at a current density of 0.4 A g^−1^. C,D) CV and galvanostatic data for A‐HIC‐Na, respectively. E) Ragone plot comparison of A‐HIC‐K and A‐HIC‐Na. F) Extended cycling performance of A‐HIC‐K and A‐HIC‐Na.

The CV curves for A‐HIC‐K shown in **Figure** **6**A demonstrate highly resistive behavior as indicated by their truncated shape, and the nearly 45° slope in the anodic currents. As is shown in Figure 6B, for A‐HIC‐K there is a major *IR* drop even at a low current density of 0.4 A g^−1^. Because of such severely limited rate capability of A‐HIC‐K, it was not analyzed at higher current densities. We attribute such poor rate capability to the kinetic sluggishness of the HC anode, as documented with K/K^+^ half‐cell results. The CV and galvanostatic performance of the A‐HIC‐Na device are shown in Figure 6C,D, respectively. As shown in Figure 6C, the CV curves of A‐HIC‐Na device display a box‐like pseudocapacitive shape overlaid with redox humps due to the anode. At higher rates, the CVs and the galvanostatic profiles become more distorted, although the *IR* drop remains relatively low even at 12.8 A g^−1^. Figure 6E compares the Ragone plot of A‐HIC‐Na with the single data point obtained for A‐HIC‐Na. The specific energy and specific power values are based on the total mass of the active and inactive materials in both electrodes. The A‐HIC‐Na shows fairly good performance, superior to the data point for the A‐HIC‐K device. The A‐HIC‐K cell yields 77 Wh kg^−1^ at 2830 W kg^−1^. The A‐HIC‐Na cell shows a flat energy–power profile with 170 Wh kg^−1^ at 2800 W kg^−1^, 152 Wh kg^−1^ at 5500 W kg^−1^, 127 Wh kg^−1^ at 11 100 W kg^−1^, 93 Wh kg^−1^ at 20 880 W kg^−1^, 54 Wh kg^−1^ at 38 400 W kg^−1^, and 25 Wh kg^−1^ at 67 200 W kg^−1^.

Figure 6F compares cycling stability of the A‐HIC‐K and A‐HIC‐Na devices. The A‐HIC‐Na cell survives 10 000 cycles with 70% capacity retention. Its cycle 1 CE is 74%, cycle 2 CE is 83%, cycle 10 CE is 95%, cycle 50 CE is 98%, and close to 100% CE (within measurement accuracy) afterward. The A‐HIC‐K cell starts on cycle 1 CE at 65%, cycle 2 CE at 77%, cycle 10 CE is 88%, cycle 50 CE is 84%, and around 87% afterward. This indicates that with K, there is more irreversible ion trapping at every cycle than with Na. It may be observed that the A‐HIC‐K device cycles quite poorly. Likely this is a direct outcome of the inferior rate capability of the anode, which progressively fails to potassiate/depotassiate even at 0.4 A g^−1^. During early‐stage cycling, the K ions become trapped in the hard carbon, impeding additional charging–discharging. Since capacity fading occurs early on, we do not believe that this is a mechanical degradation effect akin to what is observed with extended cycling of hard carbons with Na or Li.

The above energy–power values, like the vast majority of HIC and conventional ultracapacitor literature, are based on active material loading. Without knowing the final full‐cell design (e.g., number of layers in a multistack pouch, cylindrical vs prismatic), it is difficult to estimate the device‐level characteristics, or to provide meaningful comparisons with conventional LIBs. In fact, NIC and KIC cells containing the same active materials may yield quite different performance characteristics depending on the packaging. For example, it is common to assume a factor of 4 conversion from active material–based to device‐based specific energy values. This gives A‐HIC‐Na device maximum energy in the 40 Wh kg^−1^ range, which is approximately five to six times lower than that of the state‐of‐the‐art LIB. However, a factor of 4 actually assumes commercial ultracapacitor‐equivalent mass loading of 6–10 mg cm^−2^. A more scientific literature‐representative loading is in the range of 1 mg cm^−2^. This would increase the conversion factor from 4 to 10 or even higher, making the disparity between HICs and LIBs even greater.

The energy density (volumetric) disadvantage of an HIC would be even worse. Most commercial ultracapacitor devices are not mass loading limited but rather electrode thickness limited. Many commercial and near‐commercial ultracapacitors possess electrodes in the 65 μm thickness range, this being the practical processing limit for wet slurry processing. One would expect a commercial all‐carbon HIC to have a comparable electrode thickness limitation. A commercial activated carbon such as YP‐50 (Kuraray) gives an electrode density of ≈650 mg cm^−3^ resulting in a mass loading of 3.25–6.5 mg cm^−2^ (measured in‐house). Conversely, an electrode based on a high surface area, loosely packed nanocarbon such various reduced graphene oxide materials or templated carbons will be far lower, in the 200–300 mg cm^−3^ range (also measured in‐house). LIBs employ ceramic cathodes and low surface area graphite anodes, both of which are much denser than the ultracapacitor and HIC electrodes. This gives LIBs a substantial volumetric advantage. Batteries also possess an energy advantage in terms of having a relatively flat voltage profile, whereas HICs possess a triangular profile with an average voltage that is about 0.5(*V*_max_ + *V*_min_). Nevertheless, a ceramic anode–graphite cathode LIB will not cycle 100 000 times (especially with deep discharge) and will have significant difficulty charging at a rate of above 3*C*. This is where HIC devices are the most promising, not to compete directly with Li, Na, and K ion batteries in terms of energy, but to offer far higher cyclability and power. The sloping voltage profile of an HIC will also increase the fast charge safety of a device since most of the capacity is accumulated far above the equilibrium metal plating potential of 0 V versus Na/Na^+^, K/K^+^, or Li/Li^+^.

While we provide a direct Na versus K comparison, we also caution against overinterpreting these results. In our opinion, a substantial part of “good” versus “OK” performance of various electrode materials (especially in a half‐cell vs K/K^+^) is strongly linked to the relative immaturity of the K electrolytes. At this point, there are still not enough KIC or high‐rate K carbon studies so as to allow for a completely rigorous comparison of the intrinsic performance of material sets. KICs are just too new, and Na (and Li)‐inherited electrolyte and electrode paradigms are bound to be disproven. We have shown that in a half‐cell versus K/K^+^, it is the negative electrode that is highly problematic for rate and cycling life. This is likely due to the formation of a “worse” (needs to be quantified) SEI layer and greater solid‐state diffusional limitations with K versus Na. As demonstrated earlier, K does not appreciably intercalate into HC, so repeated volume changes are likely not the cause of the observed unstable SEI. Designing new classes of K carbon anode materials that offer a combination of energy, power, and cyclability remains a formidable challenge. It may be found that with standard carbonate electrolytes, this may be much more difficult to achieve with K as it is with Na or Li. More studies in this area are definitely welcome. Moreover, fast charge K ion storage mechanisms in carbons are poorly understood, and may substantially differ from the established charge storage mechanisms in comparable materials tested with Na or Li.

## 3 Conclusions

This is the first report to directly compare the properties of “classic” HC microparticle K/K^+^ half‐cell anodes and full‐cell KIC devices, with baseline Na/Na^+^ half‐cells and NICs. Two types of devices are constructed and analyzed: one is the standard HIC configuration based on a low surface area HC anode opposing a high surface area N‐rich AC cathode; and the other is a supercapacitor‐style configuration, where the AC is employed in both electrodes, with the anode:cathode mass ratio being 1:2. The bulk HC gave state‐of‐the‐art performance with Na/Na^+^ but was sluggish with K/K^+^. At all rates tested, there are major diffusional and SEI‐related limitations associated with K insertion/extraction, making it markedly inferior to Na. With K, there is a higher ion insertion–extraction overpotential, an order of magnitude higher impedance associated with the SEI layer, and drastically lower cycling life. However, the K and Na devices based on AC–AC perform on par in terms of specific energy, specific power, and cycling lifetime. The voltage window of the AC–AC devices is kept below the decomposition of the electrolyte and there is no Na or K metal, only solvated ions from the dissociated salts (KPF_6_, NaClO_4_). This allows the AC–AC cells to cycle with minimal SEI formation. We conclude that while KICs are highly novel, directly “Na‐inherited” microcarbon anodes with standard carbonate electrolytes may not be well suited for their application. Further research is needed to elucidate the core structure–chemistry–electrochemical property relations required for fast K charging into dense carbons with a range of electrolytes.

## 4 Experimental Section

Dried silk worm excrement was employed as a precursor for the high surface area N‐rich carbon, termed “AC”, i.e., activated carbon. The material was precarbonized at 400 °C and allowed to cool. The partially carbonized material was then mixed with an aqueous solution of KOH (Adamas‐beta) in a mass ratio of 3:1, followed by activation at 800 °C for 100 min under N_2_ flowing at 100 mL min^−1^ in a horizontal quartz tube furnace. The slurry was then dried at 70 °C to remove the water. The precursor–KOH mixture was activated under a N_2_ flow rate of 100 mL min^−1^ in a horizontal alumina tube furnace at 800 °C. The heating rate to temperature was 5 °C min^−1^, followed by 100 min hold and then natural cooling. The obtained product was washed with 1.0 m HCl to remove the inorganic impurities, and then washed with deionized water until the sample became neutral. The product was then dried for 10 h at 70 °C. The hard carbon termed “HC” was derived from mulberry tree stems, the leaves being the silkworm feed. The stems were carbonized at 1200 °C in flowing Ar, washed with dilute hydrochloric acid and deionized water, and dried.

SEM (JSM‐65900LV, JSM‐7500F, JEOL) and TEM (JEM‐2100F, JEOL) were employed to analyze the morphology and structure of the specimens. Elemental analysis (Elementar, Vario EL III) and XPS (UlVAC‐PHI PHI 5000 VersaProbe) were employed to provide information regarding bulk chemistry and surface functional groups. The Raman spectra were collected with 532 nm excitation and 20× objective on a Thermo Nicolet Almega system. The laser power was <2 mW. XRD analyses of the prepared ACs were carried out using a Bruker‐D8 Advance X‐ray diffractometer at a scanning speed of 5° min^−1^. The textural properties were determined at 77 K using nitrogen using a JWGB Sci. & Tech JW‐BK100C sorptometer over a relative pressure range of 10^−6^ to 0.995 atm. The surface area was calculated using the BET equation based on adsorption data in the partial pressure (*P*/*P*_0_) ranging from 0.02 to 0.25. The total pore volume was determined from the amount of nitrogen adsorbed at a relative pressure of 0.98. Pore size distributions were calculated by using the DFT Plus Software, which was based on calculated adsorption isotherms for pores of different sizes. Samples were degassed at 300 °C for 600 min prior to the measurements.

Electrodes for symmetrical AC–AC‐based devices were prepared by mixing 80 wt% AC, 10 wt% Super P (conductive carbon), and 10 wt% polyvinylidene fluoride binder. This mixture was coated onto an aluminum foil and dried at 70 °C for 10 h in a vacuum oven. The mass loading of ACs on each electrode was close to 4 mg cm^−2^, which may be considered relatively high for a laboratory study, especially with the slower diffusing K ions. Asymmetric HC–AC devices were assembled with the HC as the negative electrode “anode” and AC as the positive electrode “cathode.” The mass loading ratio between the anode and the cathode was ≈1:2. Since our aim was to provide direct general comparisons rather than optimizing system performance, no electrolyte additives were employed. Electrochemical testing was done using laboratory‐grade CR2032 stainless steel coin cells at room temperature. LAND (CT2001A) workstations were employed for galvanostatic analysis, CHI760B workstations were employed for cyclic voltammetry, and CHI760B workstations were employed for EIS. EIS analysis was performed in the frequency range of 100 kHz to 10 mHz at the open‐circuit voltage with an alternate current amplitude of 5 mV.

Prior to NIC or KIC device assembly, the anodes were galvanostatically cycled as half‐cells versus Na/Na^+^ or K/K^+^, being performed three times between 2.5 and 0.01 V. In the 0.01 V terminally sodiated or potassiated state, the half‐cells were then disassembled, and the anodes incorporated into full‐cell NICs and KICs. Apart from the ions stored in the anode and dissolved in the electrolyte, no other Na^+^ or K^+^ source was present. The open‐circuit voltage of the as‐assembled and equilibrated NICs and KICs was about 2.0 and 2.2 V, respectively. The K electrolyte was 0.8 m KPF_6_ in 1:1 by volume ethylene carbonate/diethyl carbonate (EC/DEC). The Na electrolyte was 1 m NaClO_4_ in 1:1 EC/DEC. These salts and their concentrations agree well with what is commonly employed for Na and K ion battery electrolytes, as per the references provided in the Introduction.

The gravimetric energy (*E*_g_) and gravimetric power (*P*_g_) of devices were calculated according to the following equations:

(1) $$P_{g}=I\times \Delta V/m$$

(2) $$E_{g}=P\times t/3600$$

(3) $$\Delta V=\left(V_{\max}+V_{\min}\right)/2$$

where *I* is the discharge current (A), *m* is the mass of the total active and inactive materials on both electrodes (kg), *t* is the discharge time (h), *V*_max_ is the potential at the beginning of discharge after the *IR* drop, and *V*_min_ is the potential at the end of discharge. The device energy and power calculations were presented based on the weight of all materials in the two electrodes, including the inactive carbon black and binder. It needs to be pointed out that this is also a conservative approach since many studies base the performance on the weight of the active materials only, or just the anode (which is incorrect).

## Conflict of Interest

The authors declare no conflict of interest.

## Supporting information

Supplementary
