# Supplementary material for: Direct Structure–Performance Comparison of All‐Carbon Potassium and Sodium Ion Capacitors
Source: Adv Sci (Weinh). 2019 Apr 24;6(12):1802272. doi: 10.1002/advs.201802272 (PMC6662075; doi:10.1002/advs.201802272)
Supplement: Supplementary file 1 — Supplementary [file ADVS-6-1802272-s001.pdf]

Copyright WILEY-VCH Verlag GmbH & Co. KGaA, 69469 Weinheim, Germany, 2019.

Supporting Information

for *Adv. Sci.,* DOI: 10.1002/advs.201802272

Direct Structure–Performance Comparison of All-Carbon Potassium and Sodium Ion Capacitors

Ziqiang Xu, Mengqiang Wu, Zhi Chen, Cheng Chen, Jian Yang, Tingting Feng, Eunsu Paek, and David Mitlin**

Supporting information

Direct Structure - Performance Comparison of All-Carbon Potassium and Sodium Ion Capacitors

Ziqiang Xu‡, Mengqiang Wu‡, Zhi Chen‡, Cheng Chen, Jian Yang, Tingting Feng, Eunsu Paek, David Mitlin**

Prof. Z. Xu, Prof. M. Wu, Dr. Z. Chen, C. Chen, J. Yang, T. Feng, Center for Advanced Electric Energy Technologies (CAEET), School of Materials and Energy, University of Electronic Science and Technology of China, Chengdu 611731, China, mwu@uestc.edu.cn

Prof. E. Paek, Chemical & Biomolecular Engineering, Clarkson University, Potsdam, NY 13699, USA

Prof. D. Mitlin, Walker Department of Mechanical Engineering, The University of Texas at Austin, Austin, Texas 78712-1591, USA, dmitlin@clarkson.edu

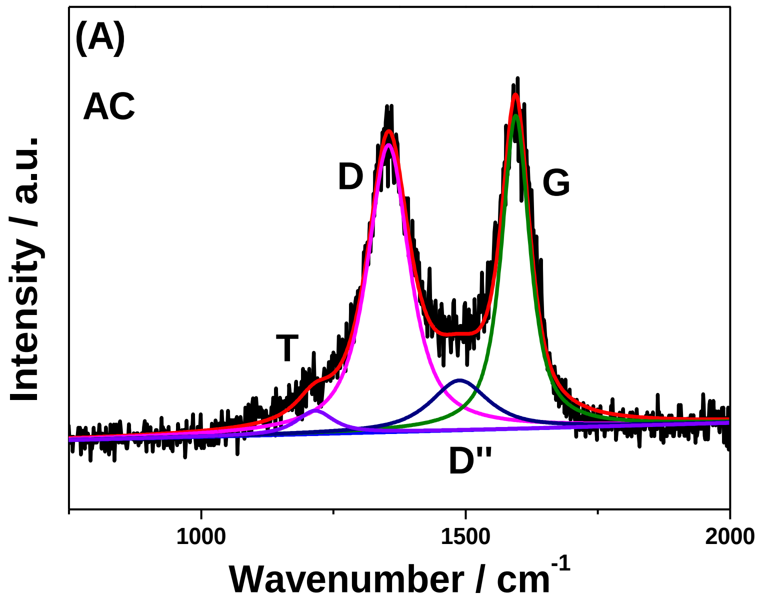

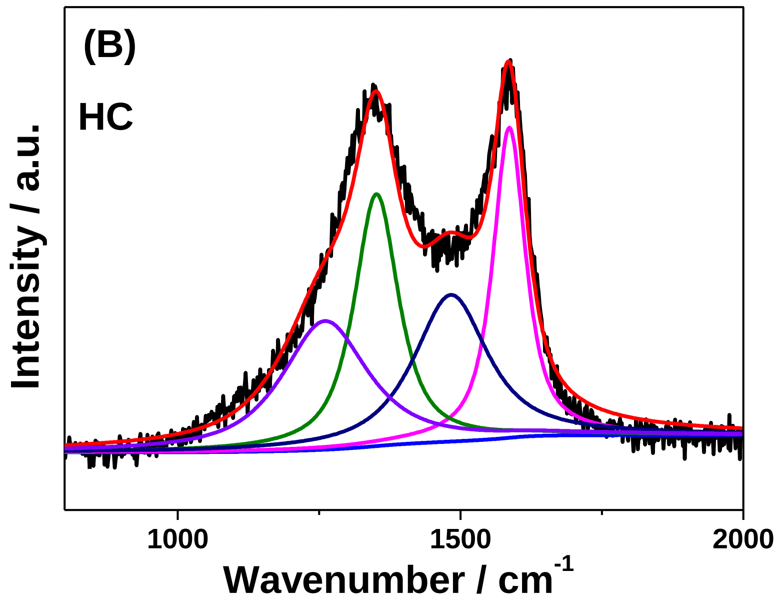

Figure S1. Fitted Raman spectra of AC (A) and HC (B).

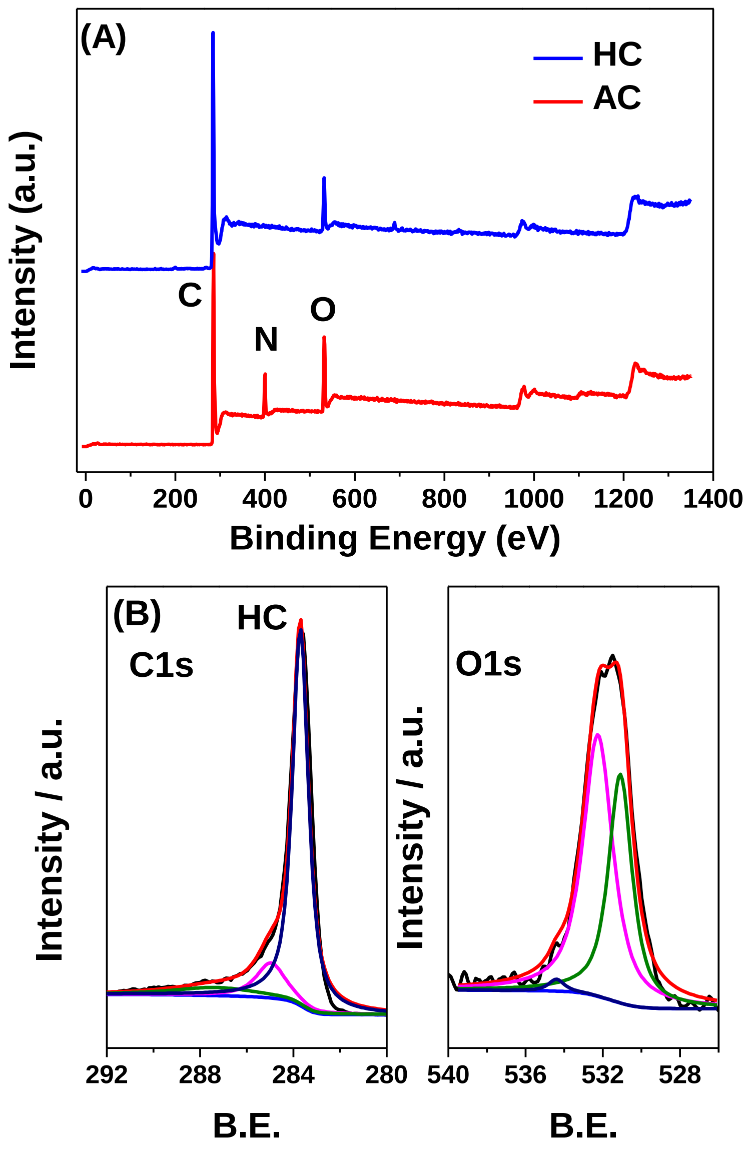

Figure S2. (A) XPS survey spectra scans for HC and AC. (B) C1s and O1s core level XPS spectra fits for HC.

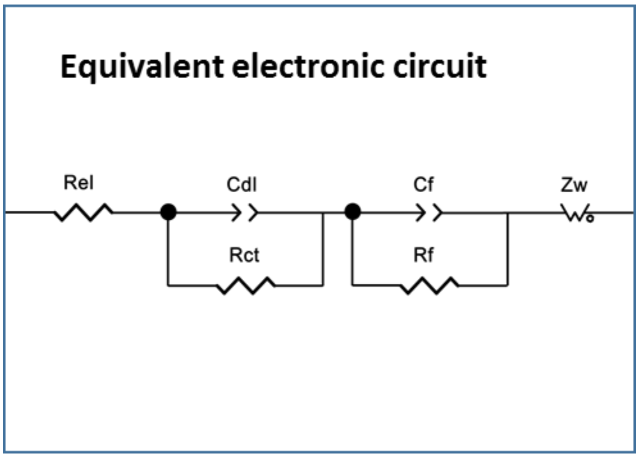

Figure S3. Equivalent electronic circuit used to simulate the EIS data.

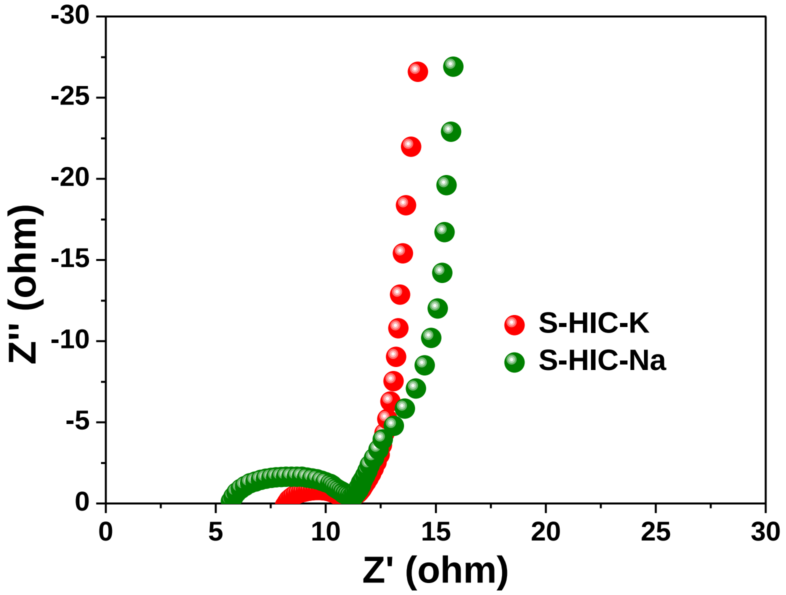

Figure S4. Experimental Nyquist Plots of S-HIC-K and S-HIC-Na.
